# PATIENT PRIORITIES CARE: THE PAST, THE PRESENT, AND THE FUTURE

**DOI:** 10.1093/geroni/igad104.1420

**Published:** 2023-12-21

**Authors:** Aanand Naik

**Affiliations:** University of Texas Health Science Center at Houston, Houston, Texas, United States

## Abstract

Healthcare based on patient goals can improve the care of older adults, particularly those with MCC. Prior studies have identified five healthcare values that guide how patients establish healthcare goals and make medical decisions: 1) Self-sufficiency, 2) Enjoyment, 3) Connection, 4) Balancing quality and length of life, 5) Engagement in care. The Patient Priorities Care (PPC) approach is an evidence-based approach that allows patients to identify their healthcare values, or what matters most to them, and identify one thing, the most important goal they want to address based on their healthcare preferences. We have demonstrated that PPC is feasible and results in less burdensome care, including fewer medications and referrals to specialists. In the VA system, we have also shown that PPC helps clinicians recommend home and community services that are aligned with the patient’s priorities. To enhance its relevance for Veterans, African Americans, and Hispanics, a deliberate effort to adapt PPC has been used at different institutions. We believe that evidence-based interventions like PPC can help address health disparities by: 1) improving communication between healthcare providers and patients with different beliefs, race, ethnicity, and culture; and 2) aligning prevention and timely treatment with the health priorities of older adults with MCC. We will review what we have learned from PPC thus far. We will then describe current efforts to implement PPC in a more effective and sustainable manner and disseminate it in diverse healthcare systems. Finally, we will share our vision moving forward and opportunities for collaboration and growth.

